# 1-Nitro-9*H*-carbazole

**DOI:** 10.1107/S1600536813032704

**Published:** 2013-12-07

**Authors:** Paul Kautny, Berthold Stöger

**Affiliations:** a Institute for Applied Synthetic Chemistry, Division Organic Chemistry, Vienna University of Technology, Getreidemarkt 9/163-OC, A-1060 Vienna, Austria; b Institute for Chemical Technologies and Analytics, Division Structural Chemistry, Vienna University of Technology, Getreidemarkt 9/164-SC, A-1060 Vienna, Austria

## Abstract

In the title mol­ecule, C_12_H_8_N_2_O_2_, the nitro group is tilted slightly with respect to the carbazole moiety [angle between the least-squares planes = 4.43 (9)°]. In the crystal, the mol­ecules are connected *via* pairs of N—H⋯O hydrogen bonds into dimers with -1 symmetry. The dimers in turn are arranged into layers parallel to (10-1).

## Related literature   

For the applications of aryl­amines as electron donors, see: Shirota & Kageyama (2007 ▶); Tao *et al.* (2011 ▶); Yook & Lee (2012 ▶); Kautny *et al.* (2014 ▶). For the synthesis of the catalyst (NHC)Pd(all­yl)Cl, see: Marion *et al.* (2006 ▶).
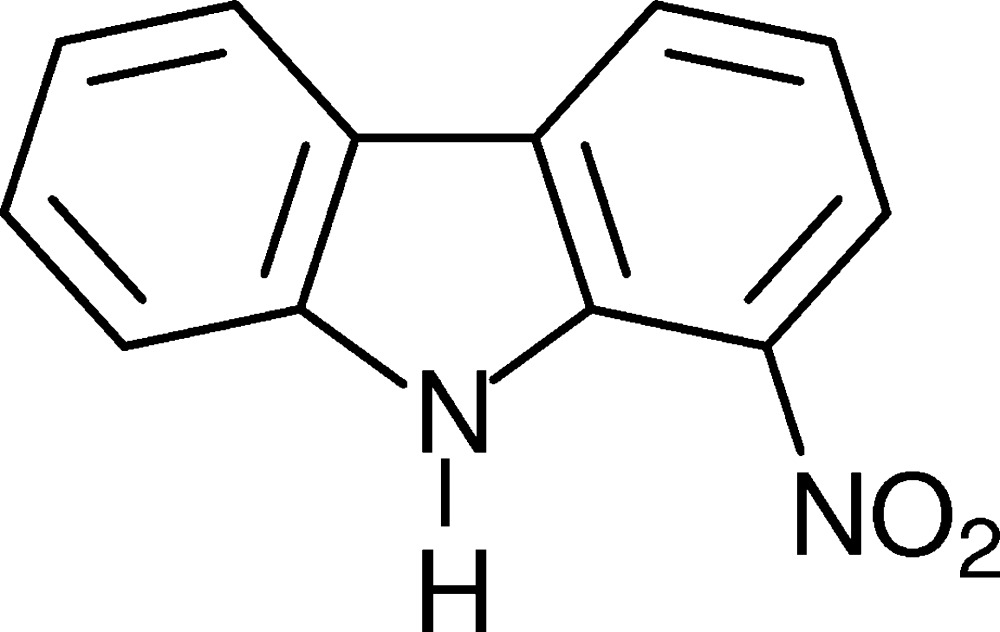


## Experimental   

### 

#### Crystal data  

C_12_H_8_N_2_O_2_
*M* *_r_* = 212.2
Monoclinic, 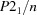

*a* = 10.4400 (3) Å
*b* = 5.3148 (2) Å
*c* = 17.2638 (6) Å
β = 99.7460 (16)°
*V* = 944.08 (6) Å^3^
*Z* = 4
Mo *K*α radiation
μ = 0.11 mm^−1^
*T* = 100 K
0.76 × 0.42 × 0.20 mm

#### Data collection  

Bruker Kappa APEXII CCD diffractometer
Absorption correction: multi-scan (*SADABS*; Bruker, 2013 ▶) *T*_min_ = 0.95, *T*_max_ = 0.98
30130 measured reflections
2794 independent reflections
2397 reflections with *I* > 3σ(*I*)
*R*_int_ = 0.021

#### Refinement  

*R*[*F* ^2^ > 3σ(*F* ^2^)] = 0.040
*wR*(*F* ^2^) = 0.068
*S* = 1.89
2794 reflections
177 parameters
All H-atom parameters refined
Δρ_max_ = 0.32 e Å^−3^
Δρ_min_ = −0.21 e Å^−3^

### 

Data collection: *APEX2* (Bruker, 2013 ▶); cell refinement: *SAINT-Plus* (Bruker, 2013 ▶); data reduction: *SAINT-Plus*; program(s) used to solve structure: *SUPERFLIP* (Palatinus & Chapuis, 2007 ▶); program(s) used to refine structure: *JANA2006* (Petříček *et al.*, 2006 ▶); molecular graphics: *ATOMS* (Dowty, 2006 ▶); software used to prepare material for publication: *publCIF* (Westrip, 2010 ▶).

## Supplementary Material

Crystal structure: contains datablock(s) global, I. DOI: 10.1107/S1600536813032704/ff2123sup1.cif

Structure factors: contains datablock(s) I. DOI: 10.1107/S1600536813032704/ff2123Isup2.hkl

Supporting information file. DOI: 10.1107/S1600536813032704/ff2123Isup3.cml

Additional supporting information:  crystallographic information; 3D view; checkCIF report

## Figures and Tables

**Table 1:** Hydrogen-bond geometry (Å, °)

*D*—H⋯*A*	*D*—H	H⋯*A*	*D*⋯*A*	*D*—H⋯*A*
N1—H*n*1⋯O1^i^	0.857 (13)	2.159 (13)	2.9940 (10)	164.6 (14)

Symmetry code: (i) 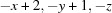.

**Fig. 1.**
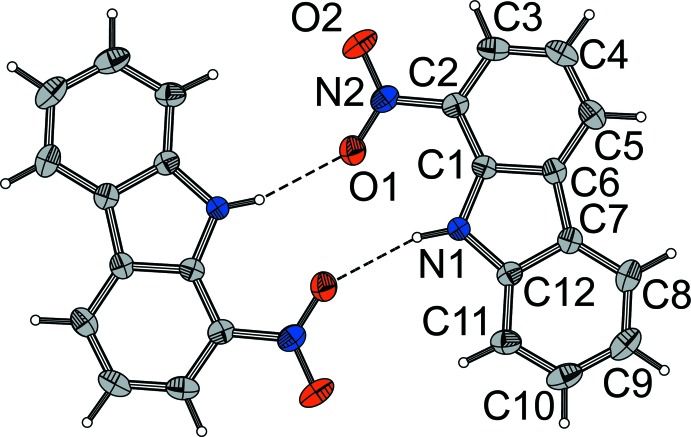
Dimer of (I) molecules related by inversion and connected via N—O···H hydrogen bonds. C, N and O atoms are represented by grey, blue and red ellipsoids drawn at the 75% probability level, H atoms by white spheres of arbitrary radius. Hydrogen bonds are indicated by dashed lines.

**Fig. 2.**
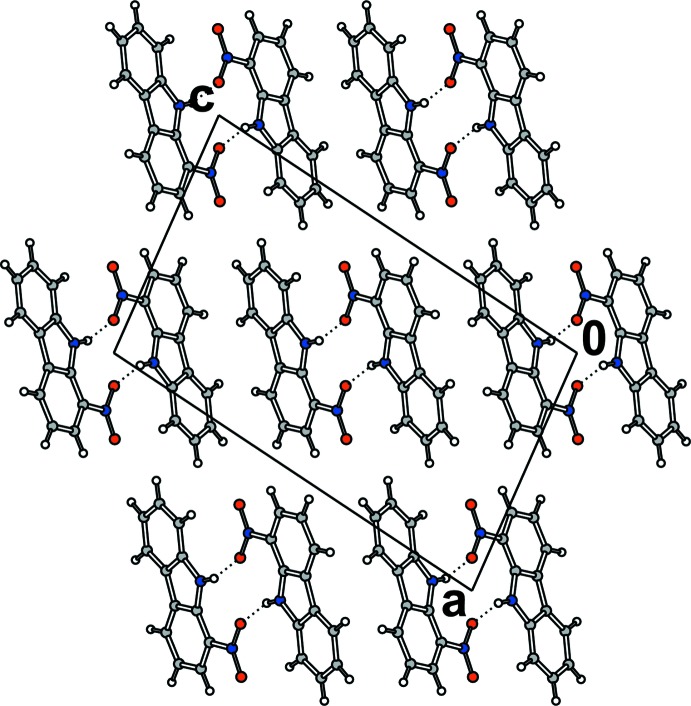
The crystal structure of (I) viewed down [010]. Atoms are represented by spheres of arbitrary radius. Colour codes as in Fig. 1.

## supplementary crystallographic information

### 1. Comment

In the last years, arylamines have been widely employed as electron donors in materials for organo-electronic applications as for example organic light emitting diodes (OLEDs) (Shirota and Kageyama, 2007; Tao *et al.*, 2011; Yook and Lee, 2012). In the course of systematic investigations of the impact of planarized arylamine moieties on the photo-physical and electro-chemical properties of bipolar host materials for phosphorescent OLEDs (Kautny, *et al.*, 2013), we synthesized the title compound 1-nitro-9*H*-carbazole (I) starting from 2-bromo-*N*-(2-nitrophenyl)benzenamine. Single crystals of (I) grown from CDCl_3_ were subjected to X-ray diffraction for structure elucidation. To our knowledge, (I) is the first structurally characterized nitrocarbazole.

(I) crystallizes in the space group *P*2_1_/*n* with one molecule in the asymmetric unit. As expected, due to the conjugated π-electron system, the molecule is virtually flat. The largest distance from the least-squares (LS) plane defined by the atoms of the carbazole ring (N1, C1–C12) is only 0.1420 (10) Å, as observed for the O1 atom of the nitro group. The N2 atom of the nitro group is located at 0.0512 (10) Å from the plane, all other atoms are closer than 0.04 Å. The plane defined by the three atoms of the nitro group (N2, O1, O2) is inclined to the LS plane of the carbazole ring by 4.43 (9)°.

The molecules are connected *via* medium-strength N—H···O hydrogen bonds (N···O: 2.9940 (10) Å) to dimers with 1 symmetry (Fig. 1). The packing of the dimers, on the other hand, is solely controlled by van-der-Waals interactions. The dimers are arranged into distinct crystallo-chemical layers parallel to (101), whereby adjacent dimers are related by the *n*-glide (Fig. 2).

### 2. Experimental

2-Bromo-*N*-(2-nitrophenyl)benzenamine (147 mg, 0.50 mmol, 1 eq.), K_2_CO_3_ (138 mg, 1 mmol, 2 eq.) and (NHC)Pd(allyl)Cl (Marion *et al.*, 2006) (6 mg, 10 µmol, 2 mol%) in DMAc (2.5 ml) were heated at 140 °C for 150 h in a capped vial using a heating block. After cooling the reaction mixture was poured on water and extracted with Et_2_O. The combined organic layers were dried over anhydrous Na_2_SO_4_ and concentrated under reduced pressure. Column chromatography (light petroleum:CH_2_Cl_2_ 75:25 → 60:40) yielded 1-nitro-9*H*-carbazole (18 mg, 0.08 mmol, 17%) as a yellow solid. ^1^H NMR (200 MHz, CDCl_3_): δ = 10.01 (bs, 1H), 8.37–8.31 (m, 2H), 8.10 (d, J=7.8 Hz, 1H), 7.60–7.50 (m, 2H), 7.39–7.26 (m, 2H) p.p.m.. ^13^C NMR (50 MHz, CDCl_3_): δ = 139.7 (*s*), 133.7 (*s*), 132.1 (*s*), 127.7 (*d*), 127.5 (*d*), 127.4 (*s*), 122.2 (*s*), 121.9 (*d*), 121.2 (*d*), 120.6 (*d*), 118.7 (*d*), 111.6 (*d*) p.p.m.. Crystals suitable for single-crystal diffraction were grown by slow evaporation of a CDCl_3_ solution.

### 3. Refinement

The structure was refined against *F* values using the Jana2006 software package (Petříček *et al.*, 2006). The non-H atoms were located in the electron density map obtained by charge-flipping and refined with anisotropic displacement parameters. The H atoms were located in difference Fourier maps and freely refined.

### Crystal data

**Table:**

C_12_H_8_N_2_O_2_	*F*(000) = 440
*M**_r_* = 212.2	*D*_x_ = 1.493 Mg m^−^^3^
Monoclinic, *P*2_1_/*n*	Mo *K*α radiation, λ = 0.71073 Å
Hall symbol: -P 2yn	Cell parameters from 17802 reflections
*a* = 10.4400 (3) Å	θ = 2.4–30.1°
*b* = 5.3148 (2) Å	µ = 0.11 mm^−^^1^
*c* = 17.2638 (6) Å	*T* = 100 K
β = 99.7460 (16)°	Plate, dark yellow
*V* = 944.08 (6) Å^3^	0.76 × 0.42 × 0.20 mm
*Z* = 4	

### Data collection

**Table:**

Bruker Kappa APEXII CCD diffractometer	2794 independent reflections
Radiation source: X-ray tube	2397 reflections with *I* > 3σ(*I*)
Graphite monochromator	*R*_int_ = 0.021
ω and φ scans	θ_max_ = 30.2°, θ_min_ = 2.1°
Absorption correction: multi-scan (*SADABS*; Bruker, 2013)	*h* = −14→14
*T*_min_ = 0.95, *T*_max_ = 0.98	*k* = −7→7
30130 measured reflections	*l* = −24→24

### Refinement

**Table:**

Refinement on *F*	0 constraints
*R*[*F*^2^ > 2σ(*F*^2^)] = 0.040	All H-atom parameters refined
*wR*(*F*^2^) = 0.068	Weighting scheme based on measured s.u.'s *w* = 1/(σ^2^(*F*) + 0.0009*F*^2^)
*S* = 1.89	(Δ/σ)_max_ = 0.015
2794 reflections	Δρ_max_ = 0.32 e Å^−^^3^
177 parameters	Δρ_min_ = −0.21 e Å^−^^3^
0 restraints	

### Fractional atomic coordinates and isotropic or equivalent isotropic displacement parameters (Å^2^)

**Table:**

	*x*	*y*	*z*	*U*_iso_*/*U*_eq_	
O1	0.89295 (6)	0.40415 (13)	0.03186 (4)	0.0210 (2)	
O2	0.72187 (8)	0.32391 (14)	0.08415 (4)	0.0262 (2)	
N1	0.94574 (7)	0.15206 (14)	−0.09492 (4)	0.0148 (2)	
N2	0.79470 (7)	0.27907 (15)	0.03678 (4)	0.0172 (2)	
C1	0.84182 (8)	0.02688 (16)	−0.07504 (5)	0.0136 (2)	
C2	0.76585 (8)	0.07065 (16)	−0.01702 (5)	0.0155 (2)	
C3	0.66091 (8)	−0.08542 (18)	−0.01060 (5)	0.0190 (3)	
C4	0.62951 (9)	−0.28368 (19)	−0.06284 (6)	0.0210 (3)	
C5	0.70463 (9)	−0.33376 (17)	−0.12071 (6)	0.0184 (3)	
C6	0.81094 (8)	−0.18269 (16)	−0.12662 (5)	0.0145 (2)	
C7	0.90505 (8)	−0.17966 (16)	−0.17915 (5)	0.0148 (2)	
C8	0.92934 (10)	−0.33696 (17)	−0.24006 (5)	0.0193 (3)	
C9	1.03157 (10)	−0.27687 (19)	−0.27885 (6)	0.0226 (3)	
C10	1.10889 (9)	−0.06296 (19)	−0.25785 (6)	0.0222 (3)	
C11	1.08747 (9)	0.09238 (17)	−0.19688 (6)	0.0187 (3)	
C12	0.98542 (8)	0.03034 (16)	−0.15802 (5)	0.0145 (2)	
H11	1.1422 (11)	0.232 (3)	−0.1779 (8)	0.028 (3)*	
H5	0.6817 (11)	−0.477 (2)	−0.1571 (7)	0.019 (3)*	
H4	0.5546 (13)	−0.390 (2)	−0.0614 (8)	0.027 (3)*	
H8	0.8730 (12)	−0.480 (2)	−0.2542 (8)	0.020 (3)*	
H9	1.0526 (14)	−0.384 (3)	−0.3211 (10)	0.039 (4)*	
H3	0.6077 (12)	−0.069 (2)	0.0296 (8)	0.026 (3)*	
H10	1.1776 (11)	−0.026 (2)	−0.2858 (7)	0.018 (3)*	
Hn1	0.9851 (12)	0.275 (3)	−0.0690 (9)	0.029 (3)*	

### Atomic displacement parameters (Å^2^)

**Table:**

	*U*^11^	*U*^22^	*U*^33^	*U*^12^	*U*^13^	*U*^23^
O1	0.0207 (3)	0.0221 (4)	0.0196 (3)	−0.0021 (3)	0.0017 (3)	−0.0039 (3)
O2	0.0301 (4)	0.0324 (4)	0.0187 (4)	0.0068 (3)	0.0117 (3)	−0.0020 (3)
N1	0.0153 (3)	0.0140 (4)	0.0157 (4)	−0.0019 (3)	0.0041 (3)	−0.0018 (3)
N2	0.0185 (4)	0.0198 (4)	0.0132 (4)	0.0041 (3)	0.0026 (3)	0.0014 (3)
C1	0.0124 (4)	0.0148 (4)	0.0133 (4)	0.0004 (3)	0.0010 (3)	0.0016 (3)
C2	0.0141 (4)	0.0184 (4)	0.0137 (4)	0.0018 (3)	0.0017 (3)	0.0012 (3)
C3	0.0138 (4)	0.0248 (5)	0.0187 (4)	0.0012 (3)	0.0033 (3)	0.0059 (3)
C4	0.0158 (4)	0.0226 (5)	0.0242 (5)	−0.0035 (3)	0.0018 (3)	0.0064 (4)
C5	0.0177 (4)	0.0170 (4)	0.0187 (4)	−0.0024 (3)	−0.0017 (3)	0.0033 (3)
C6	0.0149 (4)	0.0141 (4)	0.0135 (4)	0.0003 (3)	−0.0005 (3)	0.0014 (3)
C7	0.0163 (4)	0.0146 (4)	0.0127 (4)	0.0016 (3)	0.0007 (3)	0.0013 (3)
C8	0.0246 (5)	0.0171 (4)	0.0151 (4)	0.0053 (3)	−0.0001 (3)	−0.0013 (3)
C9	0.0279 (5)	0.0244 (5)	0.0160 (4)	0.0102 (4)	0.0053 (3)	0.0004 (3)
C10	0.0237 (4)	0.0246 (5)	0.0201 (4)	0.0095 (4)	0.0089 (3)	0.0064 (3)
C11	0.0177 (4)	0.0182 (4)	0.0215 (4)	0.0026 (3)	0.0067 (3)	0.0045 (3)
C12	0.0152 (4)	0.0140 (4)	0.0144 (4)	0.0024 (3)	0.0030 (3)	0.0018 (3)

### Geometric parameters (Å, º)

**Table:**

O1—N2	1.2372 (11)	C5—C6	1.3875 (13)
O2—N2	1.2305 (12)	C5—H5	0.991 (11)
N1—C1	1.3651 (12)	C6—C7	1.4456 (13)
N1—C12	1.3890 (12)	C7—C8	1.3997 (13)
N1—Hn1	0.857 (13)	C7—C12	1.4068 (12)
N2—C2	1.4442 (11)	C8—C9	1.3898 (15)
C1—C2	1.3984 (13)	C8—H8	0.969 (13)
C1—C6	1.4283 (12)	C9—C10	1.4058 (14)
C2—C3	1.3935 (13)	C9—H9	0.981 (16)
C3—C4	1.3897 (14)	C10—C11	1.3854 (14)
C3—H3	0.964 (15)	C10—H10	0.951 (13)
C4—C5	1.3962 (15)	C11—C12	1.3914 (14)
C4—H4	0.967 (13)	C11—H11	0.960 (13)
			
C1—N1—C12	108.99 (7)	C1—C6—C5	120.29 (8)
C1—N1—Hn1	124.9 (10)	C1—C6—C7	106.06 (7)
C12—N1—Hn1	125.8 (10)	C5—C6—C7	133.60 (8)
O1—N2—O2	123.66 (8)	C6—C7—C8	133.90 (8)
O1—N2—C2	116.94 (8)	C6—C7—C12	106.56 (7)
O2—N2—C2	119.40 (8)	C8—C7—C12	119.52 (8)
N1—C1—C2	132.09 (8)	C7—C8—C9	118.44 (8)
N1—C1—C6	109.18 (8)	C7—C8—H8	118.6 (8)
C2—C1—C6	118.73 (8)	C9—C8—H8	122.9 (8)
N2—C2—C1	120.43 (8)	C8—C9—C10	121.06 (9)
N2—C2—C3	119.11 (8)	C8—C9—H9	120.8 (9)
C1—C2—C3	120.46 (8)	C10—C9—H9	118.1 (9)
C2—C3—C4	120.13 (9)	C9—C10—C11	121.22 (10)
C2—C3—H3	124.0 (7)	C9—C10—H10	119.1 (7)
C4—C3—H3	115.9 (7)	C11—C10—H10	119.7 (7)
C3—C4—C5	120.64 (9)	C10—C11—C12	117.41 (8)
C3—C4—H4	121.8 (9)	C10—C11—H11	123.9 (8)
C5—C4—H4	117.5 (9)	C12—C11—H11	118.6 (8)
C4—C5—C6	119.70 (8)	N1—C12—C7	109.20 (8)
C4—C5—H5	119.7 (7)	N1—C12—C11	128.47 (8)
C6—C5—H5	120.6 (7)	C7—C12—C11	122.32 (8)

### Hydrogen-bond geometry (Å, º)

**Table:**

*D*—H···*A*	*D*—H	H···*A*	*D*···*A*	*D*—H···*A*
N1—H*n*1···O1^i^	0.857 (13)	2.159 (13)	2.9940 (10)	164.6 (14)

Symmetry code: (i) −*x*+2, −*y*+1, −*z*.
